# Influence of Ceramic Debris on Osteoblast Behaviors: An *In Vivo* Study

**DOI:** 10.1111/os.12496

**Published:** 2019-08-28

**Authors:** Guo‐jing Sun, Shu‐feng Yang, Yun‐fan Ti, Guo‐dong Guo, Geng‐tao Fan, Feng‐rong Chen, Shao‐gang Xu, Jian‐ning Zhao

**Affiliations:** ^1^ Department of Orthopaedic Surgery Jinling Hospital, Nanjing University School of Medicine Nanjing China; ^2^ Department of Orthopaedic Surgery Nanjing 81th Hospital of PLA Nanjing China; ^3^ Department of Orthopaedic Surgery Xiamen 174th Hospital of PLA Xiamen China; ^4^ Department of Emergency Surgery, Zhengzhou Orthopaedic Hospital, Zhengzhou China

**Keywords:** BMP/Smads, Osteoblast, Signaling pathway, Total hip arthroplasty, Wear debris

## Abstract

**Objective:**

Wear‐induced aseptic loosening has been accepted as one of the main reasons for failure of total hip arthroplasty. Ceramic wear debris is generated following prosthesis implantation and plays an important part in the upregulation of inflammatory factors in total hip arthroplasty. The present study investigates the influence of ceramic debris on osteoblasts and inflammatory factors.

**Methods:**

Ceramic debris was prepared by mechanical grinding of an aluminum femoral head and added to cultures of MC3T3‐E subclone 14 cells at different concentrations (i.e. 0, 5, 10, and 15 μg/mL). Cell proliferation was evaluated using a Cell Counting Kit (CCK‐8), and cell differentiation was assessed by mRNA expression of alkaline phosphatase (ALP), osteocalcin (OCN), and osteopontin (OPN). In addition, cell bio‐mineralization was evaluated through alizarin red S staining, and release of tumor necrosis factor alpha (TNF‐α), interleukin‐1 beta (IL‐1β), and interleukin‐6 (IL‐6) was measured through enzyme‐linked immunosorbent assays (ELISA). Furthermore, mRNA expression of Smad1, Smad4, and Smad5 and protein expression of phosphorylated Smad1, Smad4, and Smad5 were measured by reverse transcriptase polymerase chain reaction (RT‐PCR) and western blotting.

**Results:**

The ceramic debris had irregular shapes and sizes, and analysis of the size distribution using a particle size analyzer indicated that approximately 90% of the ceramic debris was smaller than 3.2 μm (2.0 ± 0.4 μm), which is considered clinically relevant. The results for mRNA expression of ALP, OCN, and OPN and alizarin red S staining indicated that cell differentiation and bio‐mineralization were significantly inhibited by the presence of ceramic debris at all tested concentrations (*P* < 0.05, and the values decreased gradually with the increase of ceramic debris concentration), but the results of the CCK‐8 assay showed that cell proliferation was not significantly affected (*P* > 0.05; there was no significant difference between the groups at 1, 3, and 5 days). In addition, the results of ELISA, RT‐PCR, and western blotting demonstrated that ceramic debris significantly promoted the release of inflammatory factors, including TNF‐α, IL‐β, and IL‐6 (*P* < 0.05, and the values increased gradually with the increase of ceramic debris concentration), and also greatly reduced the mRNA expression of Smad1, Smad4, and Smad5 (the values decreased gradually with the increase of ceramic debris concentration) as well as protein expression of phosphorylated Smad1, Smad4, and Smad5.

**Conclusion:**

Ceramic debris may affect differentiation and bio‐mineralization of MC3T3‐E subclone 14 cells through the bone morphogenetic protein/Smad signaling pathway.

## Introduction

Total hip arthroplasty (THA) is an effective surgical procedure for treating hip disorders such as osteoarthritis, rheumatoid arthritis, and avascular necrosis, dramatically improving the quality of life of patients as a result of immediate relief from pain in the hip joint. The first tentative methods of restoration of function for patients with an arthritic hip were developed by researchers through many novel ideas, but a significant contribution to the routine success achieved today is obviously the concept of low‐friction arthroplasty proposed by John Charnley^1^. After development over more than five decades, the overall survival of THA is satisfactory. For example, the 15‐year survival of 438 733 replaced hips in the Scandinavian countries, with any revision as an endpoint, was higher than 84%^2^. Aseptic loosening is the main reason for failure of THA, which can be eventually attributed to wear debris‐induced periprosthetic bone resorption^3^. There are two types of methods currently available to fix the femoral stem; namely, the cemented method and the cementless method. Both the head–cup interface and the stem–cement interface are potential sites for the generation of wear debris^4^, ^5^, ^6^. Recently, much attention has been paid to the metallic and bone cement debris produced at the stem–cement interface following debonding of the femoral stem from the bone cement under cyclical physiological loading^7^, ^8^. Debonding of the stem–cement interface is considered inevitable for almost all stem designs, and a low‐amplitude oscillatory micromotion will occur at this interface, resulting in fretting wear and the corresponding formation of wear debris. The increasing significance of the stem–cement interface to a certain degree may be due to the great wear reduction achieved by the use of metal‐on‐metal and ceramic‐on‐ceramic articulations for the head–cup interface^9^, ^10^.

At present, the biotribo‐acoustics issue has been reported in the literature as a major concern with regard to the ceramic‐on‐ceramic articulation for THA^11^. However, it is considered that ceramic wear debris is generated inevitably during the long‐term *in vivo* service of the hip prosthesis in the human body^12^, ^13^. It has been indicated that ceramic wear debris provokes an upregulation of tumor necrosis factor alpha (TNF‐α)^14^, an inflammatory cytokine that stimulates osteoclastogenesis while simultaneously inhibiting osteoblast functioning^15^. It is generally accepted that bone reconstruction cannot be restored successfully without the involvement of osteoblasts, even if osteoclastogenesis is inhibited^16^. However, a systematic review of previously published studies indicates that a detailed investigation of osteoblast behaviors with the presence of ceramic debris is lacking. Consequently, it is crucial to gain an insight into osteoblast behaviors exposed to different concentrations of ceramic debris, which is the primary aim of the present study.

In the present study, we performed a series of *in vitro* tests to investigate the influence of ceramic debris on the osteoblast behaviors of MC3T3‐E subclone 14 cells in THA. First, we prepared ceramic debris using an aluminum femoral head by mechanical grinding. The morphology, size distribution, and phase composition features of the ceramic debris were sequentially evaluated to confirm its feasibility for the following tests. Subsequently, the mouse osteoblast‐like MC3T3‐E subclone 14 cells were co‐cultured with different concentrations of ceramic debris (0, 5, 10, and 15 μg/mL) in the incubation at an atmosphere of 37°C and 5% CO_2_. The cell proliferation behavior was evaluated by Cell Counting Kit (Ruian Bio Technologies Co., Ltd, Shanghai, China) at 1, 3, and 5 days, with the optical density being measured at an excitation wavelength of 450 nm. The cell differentiation was characterized by mRNA expression of alkaline phosphatase (ALP), osteocalcin (OCN), and osteopontin (OPN) at 3 days. The cell bio‐mineralization was evaluated by alizarin red S staining at 5 days. In addition, the release of typical tumor necrosis factors such as alpha (TNF‐α), interleukin‐1 beta (IL‐1β), and interleukin‐6 (IL‐6) was measured using enzyme‐linked immunosorbent assays (ELISA) at 3 days. Furthermore, the mRNA expression levels of Smad1, Smad4, and Smad5 and protein expression levels of phosphorylated Smad1, Smad4, and Smad5 were measured by reverse transcriptase polymerase chain reaction (RT‐PCR) and western blotting at 3 days. Thes abovementioned *in vitro* tests were carried out to investigate the potential signaling pathway of the ceramic debris affecting the osteoblast behaviors of MC3T3‐E subclone 14 cells. Finally, the data were processed for statistical analysis of significant difference.

Based on the completion of these *in vitro* tests, it is anticipated that the influence of ceramic debris on the osteoblast behaviors of MC3T3‐E subclone 14 cells can be determined, which has great value for both research and clinical relevance: (i) the effect of ceramic debris concentration on the osteoblast behavior; (ii) the potential signaling pathway for the regulation; and (iii) the importance of reducing generation of wear debris for ceramic‐on‐ceramic THA.

## Materials and Methods

### *Preparation of Ceramic Debris and Characterization*

Ceramic debris was prepared by mechanical grinding of an aluminum femoral head (Johnson & Johnson, NJ, USA; this third generation of femoral head is used in THA) using a QM‐3SP2 ball mill (Nanjing NanDa Instrument Plant, China), and then filtered by a 400‐mesh screen mesh to remove debris of larger sizes. The size distribution and morphology of the ceramic debris obtained were characterized using an HELOS/OASIS dynamic light scattering (DLS) particle size analyzer (Sympatec GmbH, Germany) and an S‐3400N scanning electron microscope (Hitachi, Japan). In addition, phase composition analysis of the ceramic debris was performed using a D8 Advance X‐ray diffractometer (XRD, Bruker, Germany) with a scanning angle of 2θ from 10° to 70°.

### *Cell Culture*

Mouse osteoblast‐like MC3T3‐E subclone 14 cells were purchased from the Type Culture Collection of the Chinese Academy of Sciences (Shanghai, China) and cultured in α‐minimal essential medium supplemented with 10% fetal bovine serum, 100 U/mL penicillin, and 100 mg/mL streptomycin. The cultured cells were placed in a CO_2_ incubator (Thermo Electron, USA) with an atmosphere of 100% humidity at 37°C and 5% CO_2_ and replaced every 3 days.

At 80% confluency, the MC3T3‐E subclone 14 cells were digested by 0.25% trypsin‐1 mmol/L ethylene diamine tetraacetic acid and seeded in 96‐well culture plates at a concentration of 5 × 10^3^ cells per 200 μL culture medium in each well. Subsequently, the ceramic debris (endotoxin concentration less than 0.1 EU/mL: treated by 3 h baking at 250°C and subsequent exposure with ultraviolet radiation; examined by using an endotoxin kit and optical adsorption at 545 nm) was added to the culture medium at final concentrations of 0 (as control), 5, 10, and 15 μg/mL. The culture plates were gently transferred to the CO_2_ incubator for incubation at 37°C in 5% CO_2_.

### *Osteoblast Proliferation, Differentiation, and Bio‐mineralization*

Cell proliferation was determined using the Cell Counting Kit (CCK‐8) method. After culturing for 1, 3, and 5 days, 10 μL CCK‐8 solution was added to each well of a 96‐well culture plate, followed by further incubation for 1 h. The optical density of the solution was measured using an MK3 enzyme labeling instrument (Thermo Electron, USA) at an excitation wavelength of 450 nm. In addition, the MC3T3‐E subclone 14 cells cultured with different concentrations of ceramic debris (0, 5, 10, and 15 μg/mL) in the culture medium for 3 days were collected, and the total RNA was extracted using the Trizol Reagent Kit (Invitrogen, USA). Primers for ALP, OCN, and OPN were designed (Table 1), and mRNA expression of these osteogenic genes was measured by RT‐PCR to evaluate cell differentiation capability (denaturalization: 94°C, 10 s; primer annealing, 60°C, 20 s; extension, 72°C, 30 s; cycle number: 40).

**Table 1:** The primers to GAPDH, ALP, OCN, OPN, Smad1, Smad4, and Smad5 designed for RT‐PCR

Genes	Primer sequence (5′ to 3′)
GAPDH	F: AGGTTGTCTCCTGCGACTTCA
	R: GAGGTCCACCACTCTGTTGCT
ALP	F: TGAGCGACACGGACAAGA
	R: GAGTGTTGTTGATGGTCCGG
OCN	F: CCAAGCAGGAGCGCAATA
	R: AATCCTGGACACGACCGGA
OPN	F: AGCAAGAAACTCTTCCAAGCAA
	R: GTGAGATTCGTCAGATTCATCCG
Smad1	F: CGTCCAACAATAAGAACCGCTTC
	R: GGCATTCCGCATACACCTCTC
Smad4	F: ACAAGTAACGATGCCTGTCTGAG
	R: AGCCACCTGAAGTCGTCCAT
Smad5	F: GAGAGTCCAGTCTTACCTCCAGT
	R: TGCGGTTCATTGTGGCTCAG

ALP, alkaline phosphatase; GAPDH, glyceraldehyde 3‐phosphate dehydrogenase; OCN, osteocalcin; OPN, osteopontin; RT‐PCR, reverse transcriptase polymerase chain reaction.

Furthermore, osteoblast bio‐mineralization was assessed through alizarin red S staining after exposure to different concentrations of ceramic debris (0, 5, 10, and 15 μg/mL) in the culture medium for 5 days.

### *Evaluation of Inflammatory Factor Release*

The release of inflammatory factors from the MC3T3‐E subclone 14 cells, including TNF‐α, IL‐1β, and IL‐6, was evaluated using commercially available ELISA kits (Mouse IL‐1 beta/IL‐1F2 DuoSet, Mouse IL‐6 DuoSet, Mouse TNF‐alpha DuoSet, R&D Systems, USA). The cells were cultured in the CO_2_ incubator with four different concentrations of ceramic debris (0, 5, 10, and 15 μg/mL) in the culture medium for 3 days, and the concentrations of inflammatory factors in the culture medium were measured based on the manufacturer's instructions for the ELISA kits.

### *Signaling Pathway Analysis*

MC3T3‐E subclone 14 cells were cultured using the same methods as mentioned above. After culturing in the CO_2_ incubator for 3 days, the total RNA was extracted using Trizol Reagent Kit. Primers to Smads (sma and mad homologs) including Smad1, Smad4, and Smad5 were designed (shown in Table 1), and mRNA expression levels of the genes were measured by RT‐PCR (denaturization: 94°C, 20 s; primer annealing, 60°C, 30 s; extension, 72°C, 30 s; cycle number: 40). In addition, protein expression levels of phosphorylated Smad1 (p‐Smad1), Smad4, and Smad5 were obtained by western blotting following standard procedures, with Smad1 and beta‐actin as the references.

### *Statistical Analysis*

All data are presented as means ± standard deviations. Statistical analyses were performed using the Statistical Package for Social Sciences software, version 12.0 (SPSS, Chicago, IL, USA). One‐way analysis of variance was used to evaluate the differences between the groups, and a *P*‐value less than 0.05 was considered statistically significant.

## Results

### *Characterization of Ceramic Debris*

**Figure 1:**
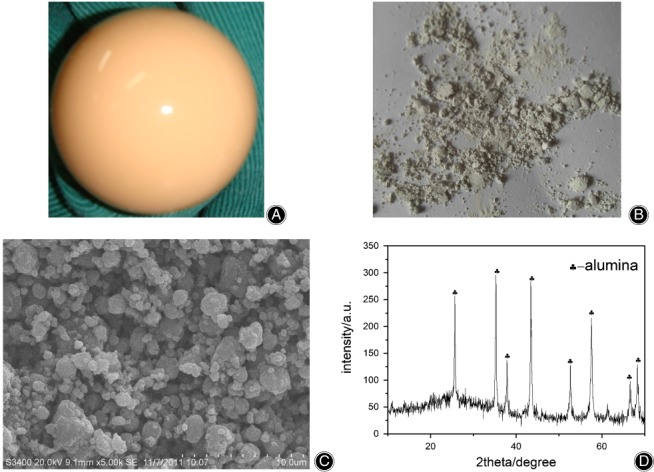
Preparation and characterization of ceramic debris: (A) original aluminum femoral head; (B) ceramic debris after mechanical grinding; (C) scanning electron microscopy characterization of ceramic debris, showing that ceramic debris featured irregular shapes and sizes; and (D) XRD characterization of ceramic debris, confirming that the main component of ceramic debris was aluminum.

Images of the original aluminum femoral head and the prepared ceramic debris along with their morphology and composition characterizations are shown in Fig. 1. The obtained scanning electron microscopy (SEM) micrographs demonstrate that the ceramic debris features irregular shapes and sizes. However, the size distribution from the particle size analyzer indicates that approximately 90% of the ceramic debris is smaller than 3.2 μm, which is considered clinically relevant^17^. In addition, the phase composition analysis obtained by XRD confirms that the main component of ceramic debris is aluminum, indicating no contamination was introduced during preparation of the ceramic debris.

### *Cell Proliferation (CCK‐8), Differentiation (Alkaline Phosphatase, Osteocalcin, and Osteopontin), and Bio‐mineralization*

**Figure 2:**
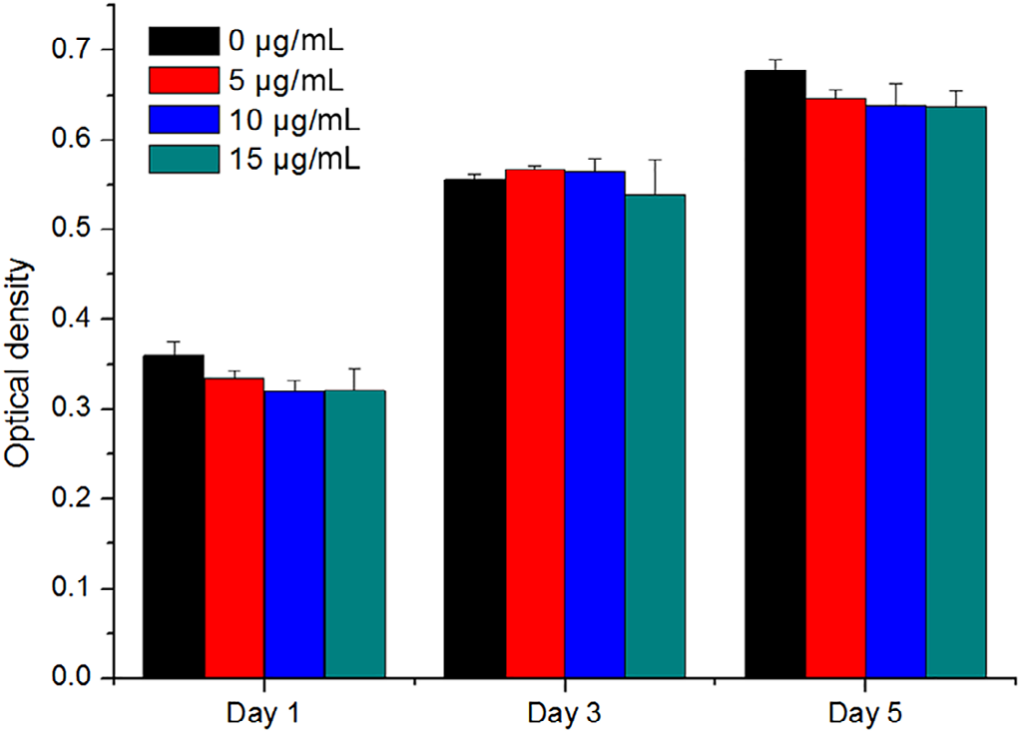
Proliferation kinetics of MC3T3‐E subclone 14 cells cultured with different concentrations of ceramic debris at 1, 3, and 5 days (*n* = 6), showing that cell proliferation was not significantly affected by ceramic debris.

**Figure 3:**
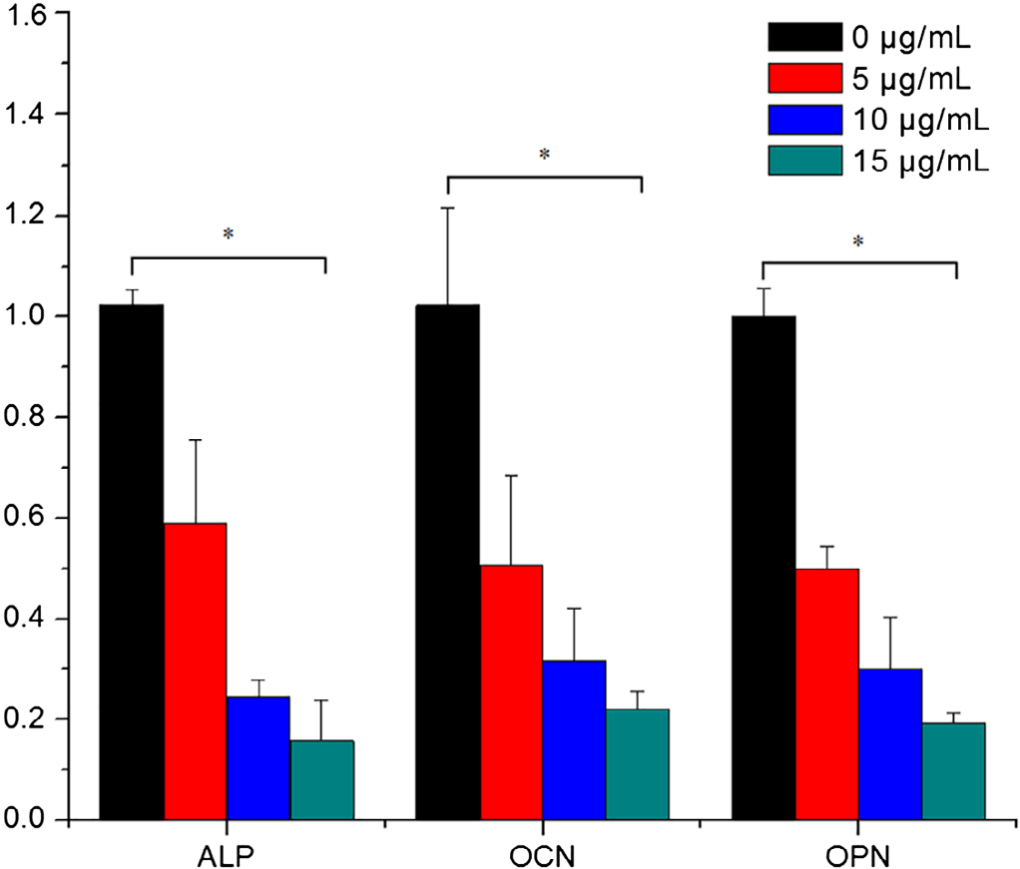
mRNA expression of alkaline phosphatase, osteocalcin, and osteopontin in MC3T3‐E subclone 14 cells after 3 days in culture with different concentrations of ceramic debris (*n* = 6), showing that cell differentiation was significantly affected by ceramic debris.

**Figure 4:**
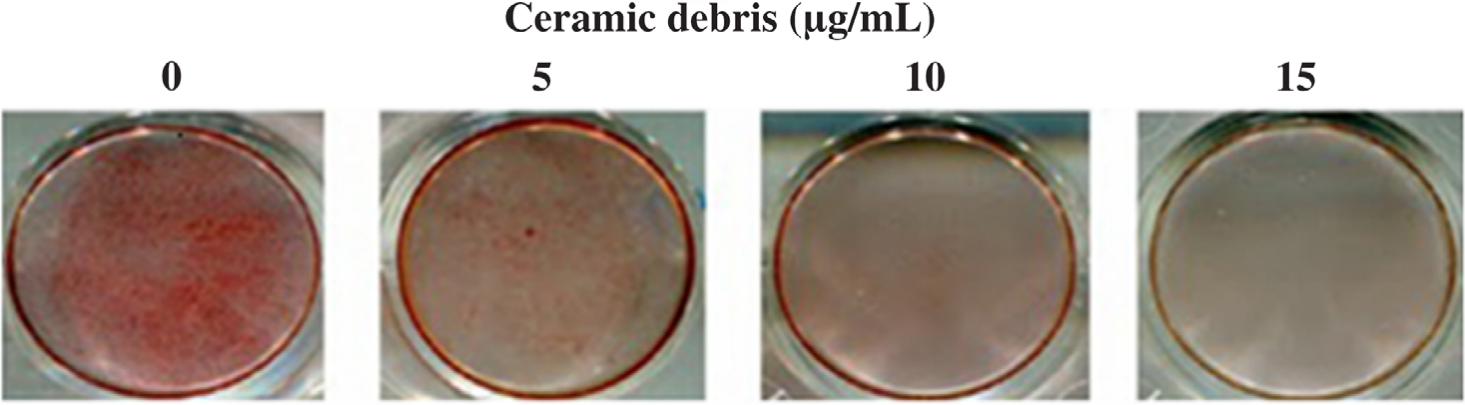
Bio‐mineralization of MC3T3‐E subclone 14 cells cultured with different concentrations of ceramic debris (0, 5, 10, and 15 μg/mL) for 5 days, which was greatly reduced with the increase in the concentration of ceramic debris.

The comparison of the cell proliferation kinetics of the MC3T3‐E subclone 14 cells cultured with different concentrations of ceramic debris (0, 5, 10, and 15 μg/mL) at 1, 3, and 5 days is shown in Fig. 2. The results indicate that there are no significant differences between the groups at any time point (*P* > 0.05), suggesting that the ceramic debris has no significant influence on the cell proliferation capability. The mRNA expression of ALP, OCN, and OPN of MC3T3‐E subclone 14 cells after 3 days in culture is presented in Fig. 3. These results indicate the mRNA expression of ALP, OCN, and OPN decreased gradually with the increase of ceramic debris concentration, and the differences between the groups with different concentrations of ceramic debris are all significant (*P* < 0.05). Therefore, it is considered that the ceramic debris impairs the differentiation capability of the MC3T3‐E subclone 14 cells, and this impairment is more distinctive with a higher concentration of the ceramic debris. Bio‐mineralization of the MC3T3‐E subclone 14 cells cultured with different concentrations of ceramic debris (0, 5, 10, and 15 μg/mL) after 5 days is shown in Fig. 4. It is clear from this figure that the number and density of the calcium nodules decreased gradually with the increase of the ceramic debris concentration, indicating that osteoblast bio‐mineralization is greatly affected by the introduction of ceramic debris in the culture medium.

### *Release of Inflammatory Factors (Tumor Necrosis Factor Alpha, Interleukin‐1 Beta, and Interleukin‐6*

**Figure 5:**
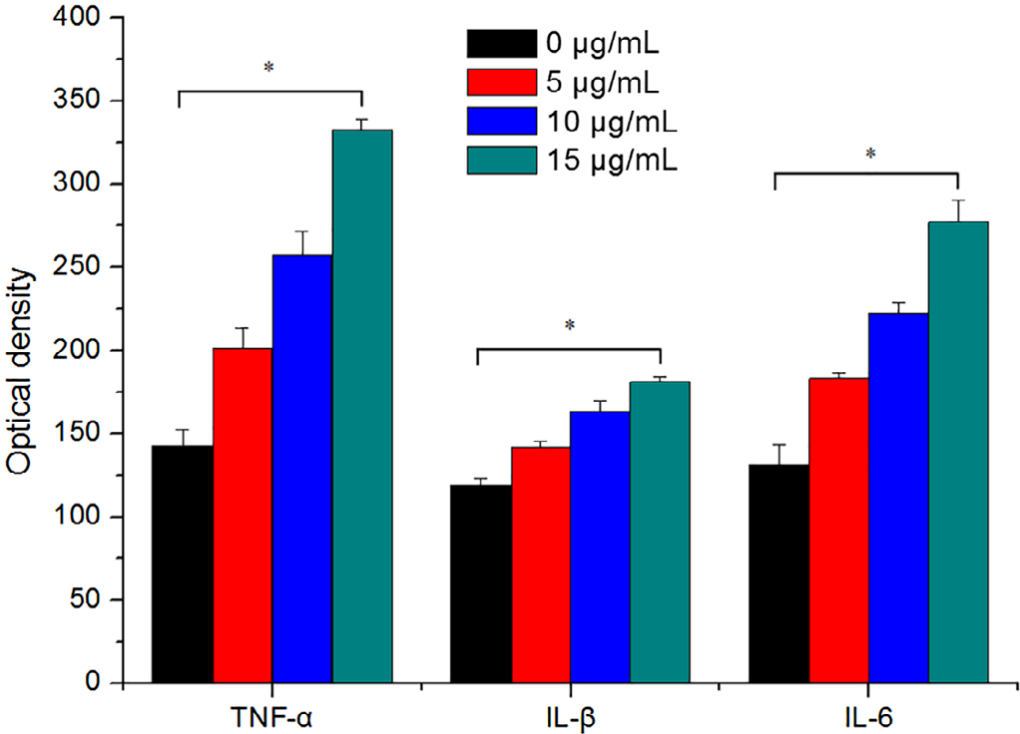
Release of tumor necrosis factor alpha, interleukin‐1 beta, and interleukin‐6 from MC3T3‐E subclone 14 cells after 3 days in culture with different concentrations of ceramic debris (*n* = 6). The release of inflammatory factors was significantly affected by ceramic debris.

The release of TNF‐α, IL‐1β, and IL‐6 by MC3T3‐E subclone 14 cells after 3 days in culture with different concentrations of ceramic debris (0, 5, 10, and 15 μg/mL) is shown in Fig. 5. The results in this figure indicate that for all three inflammatory factors, the optical density increased gradually with the increase of ceramic debris concentration, and the differences between the groups exposed to different concentrations of ceramic debris are all significant (*P* < 0.05). These results suggest that ceramic debris promotes the release of the inflammatory factors, and this effect is more obvious when the concentration of debris is higher.

### *mRNA Expression of Smad1, Smad4, and Smad5 and Protein Expression of p‐Smad1, Smad4, and Smad5*

**Figure 6:**
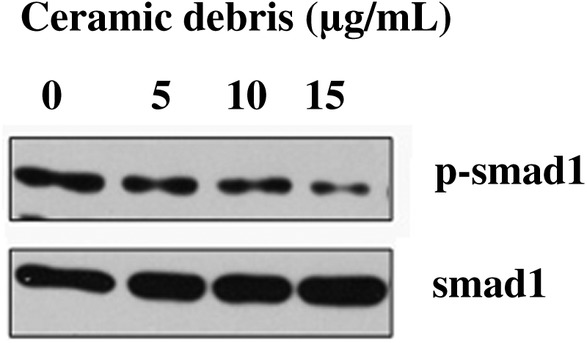
Protein expression of p‐Smad1 in MC3T3‐E subclone 14 cells after 3 days in culture with different concentrations of ceramic debris, which was greatly reduced with the increase in the concentration of ceramic debris. Note: Smad1 was used as the reference.

**Figure 7:**
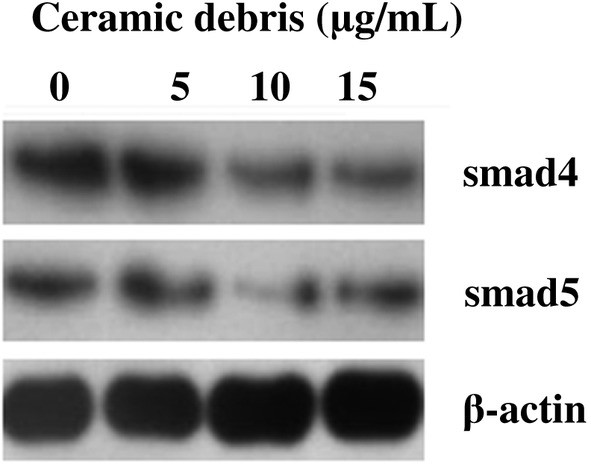
Protein expression of Smad4 and Smad5 in MC3T3‐E subclone 14 cells after 3 days in culture with different concentrations of ceramic debris, which was greatly reduced with the increase in the concentration of ceramic debris. Note: Beta‐actin was used as the reference.

The mRNA expression data for Smad1, Smad4, and Smad5 and protein expression data for p‐Smad1, Smad4, and Smad5 after 3 days in culture of the MC3T3‐E subclone 14 cells exposed to different concentrations of ceramic debris (0, 5, 10, and 15 μg/mL) are presented in Table 2 and Figs 6 and 7. The data in Table 2 demonstrate that the ceramic debris significantly inhibits the mRNA expression of Smad1, Smad4, and Smad5 (*P* < 0.05). This inhibition is more distinct with the increase of the ceramic debris concentration. In addition, the results in Figs 6 and 7 indicate that the introduction of ceramic debris results in lower levels of p‐Smad1, Smad4, and Smad5 protein expression, although the difference for Smad4 is not significant. The findings suggest that the ceramic debris may affect osteoblast differentiation and bio‐mineralization through the bone morphogenetic protein (BMP)/Smad signaling pathway.

**Table 2:** mRNA expression of Smad1, Smad4, and Smad5 in MC3T3‐E subclone 14 cells after 3 days in culture with different concentrations of ceramic debris (mean ± SD, *n* = 6)

Groups	Smad1	Smad4	Smad5
Control (0 μg/mL)	3.37 ± 0.33	10.16 ± 0.52	9.09 ± 1.80
Ceramic debris (5 μg/mL)	3.18 ± 0.47*	9.28 ± 0.46*	7.51 ± 0.63*
Ceramic debris (10 μg/mL)	2.52 ± 0.51*	8.05 ± 0.76*	6.40 ± 0.69*
Ceramic debris (15 μg/mL)	2.39 ± 0.37*	6.55 ± 0.55*	4.78 ± 0.36*

* Compared with control group (*P* < 0.05). SD, standard deviation.

## Discussion

Reducing the generation of wear debris in THA is of critical importance to further improve the longevity of this surgical procedure^18^. Previous research has investigated the influence of the wear debris generated from both the head–cup interface as well as the stem–cement interface including metallic, polyethylene and bone cement debris^19^, ^20^, ^21^, and various strategies have been proposed to protect the hip prosthesis from wear and corrosion^22^, ^23^. Wear debris generated from conventional bearing surfaces such as metal‐on‐ultra high molecular weight polyethylene is the main source of osteolysis. Ceramic debris has been neglected as great wear reduction has been achieved for the ceramic‐on‐ceramic articulation and the clinical sign for failure of ceramic hip prosthesis has been attributed to biotribo‐acoustics^24^. However, ceramic wear debris is inevitable, and the present study on effects of this inevitable ceramic debris can be important and interesting in long‐term follow‐up.

Recently, ceramic debris has attracted more attention, and the study performed by Stea *et al*. in 2012 indicated that synovial fluid microanalysis can be a useful surrogate in predicting ceramic failure, particularly when a strong presence of ceramic debris is observed^25^. In THA, a dynamic equilibrium should be established between osteoblastogenesis and osteoclastogenesis to achieve bone reconstruction^26^. In the present study, ceramic debris was prepared using mechanical grinding, and the influence of the ceramic debris on the behavior of osteoblasts in culture was investigated in terms of cell proliferation (CCK‐8), cell differentiation (ALP, OCN and OPN mRNA expression), cell bio‐mineralization, release of specific inflammatory factors (TNF‐α, IL‐1β, and IL‐6), and signaling pathway activation (BMP/Smad). The results showed that, although the presence of ceramic debris had no significant influence on cell proliferation, it significantly impaired cell differentiation and cell bio‐mineralization, promoted the release of inflammatory factors, and inhibited mRNA expression of Smad1, Smad4, and Smad5 as well as protein expression of p‐Smad1, Smad4, and Smad5 at all of the concentrations tested in the present study.

It has been shown in previous studies that cell proliferation can be inhibited by the presence of wear debris, and the influence of ceramic debris is relatively small in comparison with that of metallic debris^27^. This could be the reason why a significant difference was not observed in the proliferation of MC3T3‐E subclone 14 cells cultured with different concentrations of ceramic debris. However, in spite of being an inert biomaterial, the ceramic debris greatly reduced the mRNA expression of ALP, OCN, and OPN and promoted the release of TNF‐α, IL‐β, and IL‐6. These findings are consistent with those of the research performed by Hatton *et al*., in which alumina debris was observed to induce osteolytic cytokine production (TNF‐α) by human mononuclear phagocytes^28^. The inhibition of osteoblast cell proliferation and differentiation and promotion of osteoclastogenesis indicate that the equilibrium is disrupted by the presence of the ceramic debris, which represents one potential mechanism for the eventual aseptic loosening of the hip implant prosthesis. The BMP/Smad signaling pathway has been suggested to play an important role in the process of bone reconstruction, and osteogenic protein 1 (OP‐1) not only promotes osteoblastogenesis but also inhibits osteoclast generation^29^, ^30^. In the present study, exposure to the ceramic debris inhibited Smad1, Smad4, and Smad5 mRNA expression and p‐Smad1, Smad4, and Smad5 protein expression, which was indicative of impairment of the BMP/Smad signaling pathway. Further research will be performed to study the introduction of OP‐1 to the culture medium and its influence on the promotion of the mRNA expression of Smad1, Smad4, and Smad5 and protein expression of p‐Smad1, Smad4, and Smad5 in the MC3T3‐E subclone 14 cells.

One major limitation of the present study is that all observations were made using cultured cells *in vitro*, and an *in vivo* investigation in an animal model of osteolysis has not been performed as yet. However, it should be noted that osteolysis is a multifactorial problem, and the results of *in vivo* studies in animals cannot be used or interpreted as directly related to the condition in humans. Therefore, in the present study, we focused on investigating the behavior of MC3T3‐E subclone 14 cells (e.g. proliferation and differentiation) by adding ceramic debris to the culture medium. Further studies will be performed to validate these results when a more suitable animal model representing osteolysis is available. In addition, in future studies, we will focus on methods to promote the proliferation and differentiation of osteoblasts in the presence of ceramic debris: for example, the introduction of osteogenic protein. Finally, because debris of varying sizes may affect osteoblast behaviors differently, we will investigate potential differences in the effects of debris of varying sizes in future research.

In the present study, ceramic debris was prepared by mechanical grinding, and its influence on the osteoblast behaviors of MC3T3‐E subclone 14 cells was investigated. The presence of ceramic debris inhibited cell differentiation, cell bio‐mineralization, mRNA expression of Smad1, Smad4 and Smad5, and protein expression of p‐Smad1, Smad4 and Smad5. It promoted the release of inflammatory factors and had no significant influence on cell proliferation. The mechanism underlying these effects of ceramic debris may be related to the impairment of the BMP/Smad signaling pathway.
